# Can Social Media Be Used as a Community-Building and Support Tool among Jewish Women Impacted by Breast and Ovarian Cancer? An Evidence-Based Observational Report

**DOI:** 10.3390/healthcare10010051

**Published:** 2021-12-28

**Authors:** Catherine Dunn, Sydney Campbell, Nikoleta Marku, Adina Fleischmann, Elana Silber, Melissa Rosen, Kenneth P. Tercyak

**Affiliations:** 1 Lombardi Comprehensive Cancer Center, Washington, DC 20007, USA; cpd43@georgetown.edu (C.D.); sc2104@georgetown.edu (S.C.); nm911@georgetown.edu (N.M.); 2 Sharsheret, Teaneck, NJ 07666, USA; afleischmann@sharsheret.org (A.F.); esilber@sharsheret.org (E.S.); mrosen@sharsheret.org (M.R.)

**Keywords:** social media, cancer, education, vulnerable populations, genetic predisposition to disease, community networks

## Abstract

About 1 in 40 Ashkenazi Jewish women carry a deleterious mutation in *BRCA1/2* genes, predisposing them to hereditary breast/ovarian cancer (HBOC). Thus, efforts to prevent and control HBOC in the US must include sufficient outreach and education campaigns within and across the Jewish community. Social media (SM) is utilized in public health campaigns focused on cancer, but very little is known about the efficacy of those efforts when directed toward Jewish women at risk for (“previvors”) and affected by (“survivors”) HBOC. Here, we report on outcomes of a targeted SM campaign for this population, as led by a national not-for-profit HBOC advocacy organization. Mixed-methods data were obtained from *n* = 393 members of the community, including *n* = 20 key informants, and analyzed for engagement and satisfaction with its SM campaign and HBOC resources. Message recipients identified the SM campaign as helpful/meaningful (82%), of ‘newsworthy’ value (78%), and actionable/navigable (71%): interviews revealed that women were more likely to engage with SM if/when it featured stories relevant to their personal cancer experiences. SM is a valuable public health education tool to address the comprehensive cancer control and prevention needs of those previving and surviving with HBOC, including high-risk Jewish women.

## 1. Introduction

Approximately 1 in every 500 women in the United States has a *BRCA1/2* mutation, predisposing them to hereditary breast/ovarian cancer (HBOC) [1]. While the average woman has a 1 in 8 chance (12%) of developing breast cancer in her lifetime, women with a *BRCA1/2* mutation have an estimated 72% risk of being diagnosed with breast cancer [2]. The American Cancer Society estimates that in 2021, about 43,600 American women will die of breast cancer [3], and 13,770 will die from ovarian cancer [4]. Breast cancer is one of the most common cancers found among younger women under the age of 50, with about 11% of all breast cancers occurring in women younger than 45 [5].

Although all women in the United States should be mindful of their risk of developing breast/ovarian cancer and being a *BRCA1/2* mutation carrier, women of Ashkenazi Jewish descent are at a significantly greater risk of carrying a deleterious the *BRCA1/2* gene, compared to women of non-Ashkenazi Jewish backgrounds (1:40 vs. 1:500) [6]. There are several steps that women with a *BRCA1/2* mutation can take to decrease their chances of developing breast and/or ovarian cancer, including prophylactic surgeries (removal of breasts and/or ovaries) and receiving earlier and more frequent cancer screening tests (mammograms and breast MRIs) [7]. However, the first step to decreasing risk of breast cancer begins with awareness, knowledge, and information on the subject.

The internet is a common resource to obtain and deliver health information, including cancer support services. One study revealed that 59% of Americans have searched online for health information, with 25% reporting that they searched online to specifically understand a health condition [8]. In recent years, using social media (SM) groups has gained popularity, especially among those affected by chronic diseases. In 2011, there were 620 breast-cancer specific Facebook groups, with over one million members [9]. Like other SM platforms, Facebook provides an opportunity for online users to construct groups of individuals with similar interests. These online and SM-driven communities serve as powerful resources for the *BRCA1/2* and breast cancer-affected for the purposes of peer support, information sharing, and community building [10]. Peer support has been met with high satisfaction rates and helps some cancer patients cope with diagnosis and treatment, improves their satisfaction with medical care, increases disease knowledge, improves coping strategies, provides a sense of hope, and overall helps to alleviate the psychosocial burden of a cancer diagnosis or gene mutation [11,12,13]. Yet, prior literature lacks findings of SM’s influence as a community-building enterprise to foster connections among different constituents within the cancer community (e.g., HBOC previvors, young breast cancer survivors (YBCS), metastatic breast cancer (mBC) patients, and caregivers who are of Jewish descent).

Beginning in 2020, the COVID-19 pandemic ultimately led to the implementation of social distancing guidelines and strict lockdown measures across the globe. This has made online support services for patients with cancer and other chronic conditions even more important. Cancer patients in particular have been significantly impacted by the pandemic since many aspects of cancer care had to be deprioritized to enable health systems to respond to the pandemic [14]. Cancer patients had to adjust to living without access to many in-person healthcare services, and experienced concerns regarding delayed diagnosis, delayed surgery, missed treatments, and weakened immunity [14,15]. With some cancer patients unable to seek support from formal healthcare settings, there was an increased need for resources and support in digital spaces [16]. In many communities, this need was filled by nonprofits, social service agencies, and others staffed by community health workers and health professionals.

One organization that is committed to providing personalized support, comprehensive resources and educational outreach to constituents within the cancer community is Sharsheret. Sharsheret is a national nonprofit organization dedicated to serving the comprehensive cancer control and prevention needs of HBOC Jewish women of all backgrounds, by offering informative and supportive programming [17]. The organization’s “Link Program” consists of multiple intervention components that are designed to benefit the target population which includes: women carrying *BRCA1/2* gene mutations; women who are unaffected with HBOC but at increased genetic risk (“previvors”); women surviving with breast/ovarian cancer; and especially HBOC and YBCS, mBC patients, and cancer caregivers. The Link Program delivers services across the cancer control continuum from pre-diagnosis to diagnosis through to survivorship, by offering HBOC genetic information and education, one-on-one peer support, financial assistance, and behavioral health and emotional support for constituents and their families. Sharsheret’s mission is predominantly fulfilled through remote service delivery (via phone, website, or video chat). Sharsheret utilizes SM to promote program awareness and engagement, and to foster online connections among its members through shared experiences. Little is known about the success and impact of SM campaigns on meeting HBOC and other cancer needs.

The Centers for Disease Control and Prevention define an evaluation as a systematic method for collecting, analyzing, and using data to examine the effectiveness and efficacy of programs, as importantly, to contribute to continuous program improvement [18]. This article conducts a secondary analysis of data collected during January–March of 2020, as part of comprehensive program evaluation efforts among members of Sharsheret’s network. The findings aim to inform parallel efforts nationwide on how SM can be used as a tool to help control and prevent HBOC by providing education and psychosocial support to improve health outcomes. We sought to understand how SM content and platforms could be utilized to meet the needs of Sharsheret’s constituents. We hypothesized that Sharsheret’s SM would receive positive feedback among those who use it and hold great potential to grow the network size and engage a greater number of constituents over time.

## 2. Materials and Methods

### 2.1. Overview

Sharsheret uses SM to promote education, interventions, and resources offered through its Link Program. Sharsheret conducted an annual evaluation survey (AES) and key informant interviews (KIIs), with the goal of ascertaining satisfaction and outcome data with and about Sharsheret’s Link Program by eliciting program-specific feedback centered on the prevalence of utilization, engagement with, and benefit from Sharsheret’s resources, including its SM content. The AES aimed to determine the impact of the organization’s SM communication strategy on providing HBOC information and support for its constituents, by answering the following questions: How can Sharsheret help connect constituents online via Facebook groups? Which SM platforms are optimal for reaching constituents? Additionally, what are the perceived benefits constituents gain when engaging with Sharsheret’s SM? Evaluation measures provided specific examples of Sharsheret’s SM content in 2019 in order to gain thorough respondent-level feedback.

The AES included Likert-type response scales to measure satisfaction based on participants who ‘agree’ or ‘strongly agree’ with affirmative statements about Sharsheret SM’s ability to promote the organization’s interventions and community-serving goals. This evaluation reported herein was conducted as part of a secondary data analysis used to understand how resources provided by Sharsheret have been utilized by those they support. The study’s evaluation methods and protocol were reviewed and exempt by the Institutional Review Board at Georgetown University Medical Center.

### 2.2. Sampling and Constructs

The AES and KIIs were conducted from early January through early March of 2020. The target population for this analysis included Sharsheret’s network constituents with a focus on YBCS, mBC patients, and cancer caregivers. Survey recipients provided the organization with contact information that is maintained in an administrative database. All constituents who had provided Sharsheret with an E-Mail address at the end of the 2019 calendar year were sent an initial E-Mail invitation and follow-up reminder invitations to complete the program evaluation by clicking a URL, that linked to a new secure webpage. E-Mail invitations were sent, and responses were tracked through a secure website used to manage online surveys. Reminders were distributed to non-responding individuals on a weekly basis for up to one month post survey initiation. Constituents voluntarily chose whether or not to accept the E-Mail invitation, and participate in the survey. Upon request, printed and mailed surveys were also available to those without regular computer or internet access, and/or due to other circumstances precluding online participation. KIIs were conducted by telephone and online among *n* = 20 network stakeholders, consisting of 10 individual interviews, and two focus groups with 5 informants per group. Informants were asked parallel questions to those included in the AES, largely focused on Link Program component satisfaction and SM engagement.

### 2.3. Analyses

**Figure 1:**
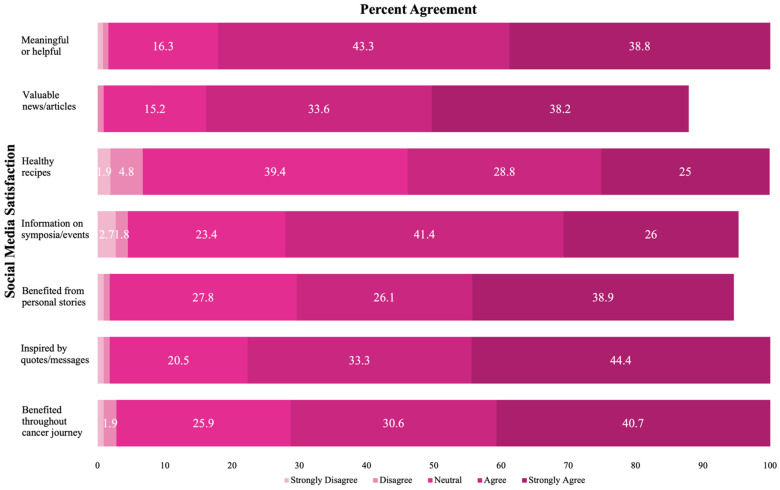
Multidimensional components of satisfaction with HBOC-related social media (*n* = 131).

The AES attempted to survey a representative population of 2000+ constituents with *n* = 393 respondents (~18% response rate). The evaluation protocol focused on the core components of the Link Program. The self-report survey included a specific SM section that inquired about participant engagement and then agreement with statements relating to Sharsheret’s SM impact on and effectiveness among its constituent base. Responses were given using a Likert-type format [strongly agree (5), agree (4), neutral (3), disagree (2), strongly disagree (1)]. Analytic cases included only those participants who engaged with Sharsheret’s SM (*n* = 131; ~33%, Figure 1). Using the statistical software program, SPSS 26.0, AES responses were analyzed. With a sample size of *n* = 131 cases, the evaluation was statistically capable of detecting small to medium effects within the data (e.g., r = 0.25) at a conventional two-tailed alpha level (0.05) and power of at least 80% to reject a hypothesis. Descriptive statistics were generated, as displayed in Table 1, featuring the demographic, clinical, and family-compositional characteristics of survey respondents. For SM utilization, we also examined its internal consistency reliability and bivariate association with age.

## 3. Results

### 3.1. Sample Characteristics

The mean age of female respondents was 52 years old. The majority reported being married (68.7%) and identified as being of Jewish descent (65.6%). Overall, 84% indicated being a cancer survivor based on the following definition: “In cancer, a person is considered to be a survivor from the time of diagnosis until the end of life” [19]. Furthermore, 23.7% self-identified as being *BRCA1/2* mutation carriers. The majority (72.3%) of respondents reported having children, with 48.6% having adult children.

### 3.2. Constituents’ Social Media Engagement and Satisfaction

Within Sharsheret’s network, 63% of respondents “frequently” used SM in their everyday lives, and 38% viewed Sharsheret’s SM platforms, which include Facebook, Instagram, and YouTube. Respondents who answered the SM-related questions shared that they utilize Facebook (87.0%), Instagram (53.7%) and YouTube (20.6%) most frequently as their Sharsheret SM platform of choice. Figure 1 displays that Sharsheret largely meets (indexed by the number of respondents who ‘agree’ or ‘strongly agree’) constituents’ expectations for its SM and online postings as being: helpful/meaningful (82.1%), of ‘newsworthy’ value (71.8%), topic relevant (53.8%), informative about upcoming events (67.4%), beneficial (65%), inspirational (77.7%), and actionable/navigable (71.3%). A summary score was derived across the 7 SM-related items, with a computed average score of 4.12 and standard deviation of 0.68 (out of a possible 5), indicating an overall positive and favorable view of Sharsheret’s SM content. Responses on this scale were highly internally consistent (Cronbach’s alpha = 0.91): YBCS were the single group most likely to follow Sharsheret on SM (44%). Age was inversely associated with SM engagement, as younger respondents engaged more often (r = −0.22, *p* = 0.01).

### 3.3. Constituents’ Facebook Group Feedback

**Figure 2:**
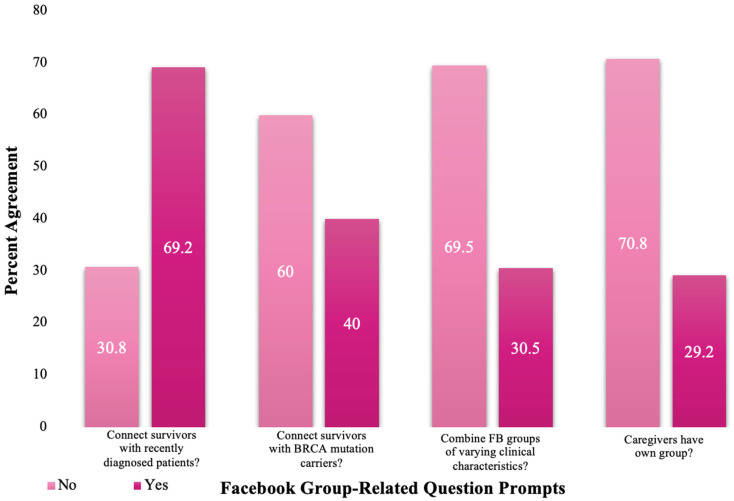
Facebook group preferences among HBOC constituents.

Sharsheret monitors two Facebook groups (one for YBCS and another for mBC patients) with the intention to educate, support, and connect individuals with shared experiences. Figure 2 depicts responses to a set of 4 yes/no Facebook group-related questions asked by the AES, including ‘would you be interested in joining a group that…’: connects cancer survivors with recently diagnosed patients (69.2% = yes); connects cancer survivors with *BRCA1/2* mutation carriers (40.0% = yes). Constituents were also asked if Facebook groups should combine those with different clinical characteristics or be maintained separately (30.5% = ‘yes’ to combine); and whether or not caregivers should be included in groups or have their own group (29.2% = ‘yes’ to caregivers having their own group.

### 3.4. Key Informant Interview Results

Qualitative KIIs provided greater insight into the AES’s Facebook group questions. Opinions on Facebook groups differed among individuals, but most respondents were excited about these Facebook groups. For the groups, most respondents felt strongly about separating the groups into caregivers and patients/those currently in treatment. Some respondents supported the idea of combining survivors and those currently battling cancer into a group to help provide hope for one another. Others even supported the idea of grouping all demographics together, survivors, currently battling and caregivers, to create a space that would help communicate the needs and fears one has to their caregiver. Feedback from these groups consistently expressed a desire for Sharsheret to attentively monitor the dialogue in the groups to ensure that marketing or advertising content is not included.

## 4. Discussion

The overall goal of this evaluation was to understand how SM can be utilized as a community-building tool to support Sharsheret’s target audience and ultimately meet the needs of those impacted by breast and ovarian cancer. This evaluation suggests that its network of previvors, YBCS, mBC patients, and cancer caregivers generally have a positive perception of Sharsheret’s SM content. Findings of this evaluation also provide insight on how constituents specifically utilize SM to meet their needs; many use Sharsheret SM to seek information, feel inspired by the stories of others, and receive overall benefits from SM engagement throughout their cancer journey. The satisfaction in Sharsheret’s digital content and knowledge of constituents’ engagement in SM groups is critical because it has the potential to positively impact health outcomes. Prior literature suggests that SM groups can alleviate the psychosocial comorbidities of cancer diagnosis by buffering against stress and providing emotional support to cancer patients [20]. Previvors and survivors of HBOC have been shown to experience psychosocial distress that can impact decision making and long-term medical management, and previous work has highlighted a need for additional support and resources for this population beyond family members and genetic counselors [21]. One study found that, despite initial hesitancy to utilize online platforms, most adults with cancer wished they had sought online support sooner [22]. This aligns with Sharsheret’s goal of providing support for those throughout their cancer journey, and not just post-diagnosis. Our evaluation shows that SM has the potential to serve as an effective community-building tool that can be used among a wide range of cancer stakeholders to alleviate the psychosocial burden of cancer.

This evaluation also highlights constituents’ opinions about specific SM platforms, particularly Facebook group use. The majority of respondents expressed interest in joining a Facebook group that connects cancer survivor with recently diagnosed patients, but the majority of respondents did not express interest in joining a Facebook group that connects cancer survivors with *BRCA1/2* mutation carriers. Overall, constituents were less inclined toward combining Facebook groups among members with different clinical characteristics: the majority believed caregivers should have their own separate Facebook group. The qualitative KIIs further highlighted constituents’ opinions regarding Facebook groups. Most respondents preferred the use of Facebook groups to connect with others impacted by HBOC. KII responses emphasized having a separate Facebook group for caregivers. However, some supported combining demographic groups to better express their needs to their caregivers. Facebook allows for wide-reaching groups which make it increasingly feasible for individuals to locate someone with a similar diagnosis or specific personal challenge [7]. Research has shown that adding breast cancer patients to an internet support group of people with the same disease improved depression, perceived stress, and cancer-related trauma scores compared to controls [23]. Similar efforts have also revealed that like-minded communities have the potential help alleviate anxiety, depression, and reactions to pain, resulting in greater emotional well-being and post-traumatic growth [23]. Furthermore, the support provided for survivors through SM can facilitate benefits such as coping and emotional connections with other survivors that may improve the well-being of cancer survivors [24]. In sum, understanding constituents’ attitudes toward SM and how they engage with SM generates insight into ways in which this technology can improve the health outcomes of those impacted by HBOC.

Like all observational reports, the present description is limited in some respects. This includes the size of the sample, its relative uniformity, and reliance on self-reporting as the primary means of data collection. This evaluation was also initiated and completed prior to widespread recognition of the COVID-19 pandemic’s reach into the United States, which likely influenced SM use as a means of socially distant and safe interaction. Future studies could improve upon this evaluation by including a larger and more diverse sample, and more in-depth assessments of psychosocial and related benefits from SM engagement. Additionally, increasing the size of and diversifying the sample would strengthen this evaluation by allowing for further subgroup analyses by SM type.

## 5. Conclusions

The purpose of this effort was to highlight the use and effectiveness of SM as a community-building tool among YBCS, mBC patients, and cancer caregivers. AES results and KII feedback reveal that there is a high level of satisfaction with Sharsheret’s SM content among those who follow and engage with the organizations SM. When reaching constituents via SM, Sharsheret provides meaningful content of newsworthy value, that helps to benefit and inspire its target audience throughout their cancer journey. Therefore, when using SM, Sharsheret is helping to meet their organization’s mission of supporting the needs of Jewish YBCS, mBC patients, and cancer caregivers. There remains opportunity for similar organizations to engage a large number of constituents in online community-building efforts through the creation of new sub-population Facebook groups.

This evaluation posits that SM can inform public health cancer outreach and education efforts to impact their target populations and diverse audiences, share new public health information about HBOC, listen and collect meaningful program feedback, and create opportunities for network members to engage directly with the host organization [9]. This evaluation distinguishes itself in the focus on SM as a tool to connect different stakeholders in the community to build greater awareness of HBOC cancer control and prevention more broadly. As the United States continues to socialize online through and beyond the COVID-19 crisis, it is imperative that community-based breast and ovarian cancer organizations continue to understand current SM use. Online media platforms provide opportunities to connect different stakeholders to share information and personal experiences that could promote their psychosocial well-being and physical health.

## Figures and Tables

**Table 1:** Characteristics of social media users among annual evaluation survey respondents (*n* = 131) *.

	Mean	SD	*n*	%
Demographics				
Age (*n* = 128)	51.8	11.9	-	-
Marital Status (*n* = 131)	-	-		
Married/Partnered	-	-	90	68.7
Separated/Divorced/Widowed/Single/Never Married	-	-	41	31.3
Religious Affiliation (*n* = 131)	-	-	-	-
Jewish Descent	-	-	86	65.6
Ashkenazi	-	-	79	60.3
Clinical Characteristics				
Among Survivors, Age at Cancer Diagnosis (*n* = 109)	54.0	51.8	-	-
Cancer Survivor (*n* = 131)	-	-	-	-
Yes	-	-	110	0.84
No	-	-	21	0.16
Cancer Status (*n* = 166)	-	-	-	-
At high risk for HBOC, *BRCA1/2* carrier	-	-	31	23.7
Recently diagnosed with breast cancer	-	-	16	12.2
Living with breast cancer	-	-	33	25.2
A breast cancer survivor	-	-	55	42.0
Recently diagnosed with ovarian cancer	-	-	0	0
Living with ovarian cancer	-	-	9	6.9
An ovarian cancer survivor	-	-	10	7.6
Other	-	-	12	9.2
Family Composition				
Respondents with Children (*n* = 130)	-	-	-	-
Yes	-	-	94	72.3
No	-	-	36	27.7
Number of Children (*n* = 130)	-	-	-	-
0	-	-	36	27.7
1–2	-	-	61	46.9
3 or more	-	-	33	25.4
Age(s) of Child(ren), in years (*n* = 129)	-	-	-	-
0–9	-	-	22	16.8
10–17	-	-	42	32.1
18+	-	-	65	49.6

* Sample sizes vary due to missing data, and because not all variables and/or levels of response within a category were applicable to all respondents. Cancer status frequency counts are greater due to the allowing of multiple responses across categories. Family composition percentages do not add-up to 100% due to households with children in multiple age categories.

## Data Availability

The data are not publicly available due to confidentiality and data protection issues involved.
